# Synthesis, crystal structure, and Hirshfeld surface analysis of 1,3-di­hydro-2*H*-benzimidazol-2-iminium 3-carb­oxy-4-hy­droxy­benzene­sulfonate

**DOI:** 10.1107/S2056989024008557

**Published:** 2024-09-06

**Authors:** Salmon Mukhammadiev, Sarvar Rajabov, Akmaljon Tojiboev, Jamshid Ashurov, Sardor Murodov, Shahlo Daminova

**Affiliations:** a Uzbekistan Japan Innovation Center of Youth, University str. 2B, Tashkent, 100095, Uzbekistan; b National University of Uzbekistan named after Mirzo Ulugbek University str 4 Tashkent 100174 Uzbekistan; c University of Geological Sciences, Olimlar str. 64, Tashkent 100125, Uzbekistan; d Institute of Bioorganic Chemistry of Academy of Sciences of Uzbekistan, Mirzo, Ulug‘bek st. 83, Tashkent 100125, Uzbekistan; e National University of Uzbekistan named after Mirzo Ulugbek University str 4 Tashkent 100174 Uzbekistan; Vienna University of Technology, Austria

**Keywords:** benzimidazolium, sulfobenzoate, crystal structure, Hirshfeld surface analysis

## Abstract

The asymmetric unit of the title salt, C_7_H_8_N_3_^+^·C_7_H_5_O_6_S^−^, comprises two 1,3-di­hydro-2*H*-benzimidazol-2-iminium cations and two 2-hy­droxy-5-sulfobenzoate anions (*Z*′ = 2). In the crystal, the mol­ecules inter­act through N—H⋯O, O—H⋯O hydrogen bonds and C—O⋯π contacts. The hydrogen-bonding inter­actions lead to the formation of layers parallel to (01). Hirshfeld surface analysis revealed that H⋯H contacts contribute to most of the crystal packing with 38.9%, followed by H⋯O contacts with 36.2%.

## Abstract 2

The asymmetric unit of the title salt comprises two 1,3-di­hydro-2*H*-benzimidazol-2-iminium cations and two 2-hy­droxy-5-sulfobenzoate anions (*Z*′ = 2). In the crystal, the mol­ecules inter­act through N—H⋯O, O—H⋯O hydrogen bonds and C—O⋯π contacts. The hydrogen-bonding inter­actions lead to the formation of layers parallel to (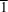
01).

## 1. Chemical context

The increasing development of benzimidazoles and their derivatives in medicine is still the subject of intensive research due to their diverse biological activities (Suku & Ravindran, 2023 ▸). Benzimidazole derivatives have anti­hypertensive, anti­allergic, anti­diabetic, anti-inflammatory, mycobacterial, anti­oxidant, anti­protozoal, anti­viral and anti­microbial effects (Dokla *et al.*, 2020 ▸; Dvornikova *et al.*, 2019 ▸; Aboul-Enein & El Rashedy, 2015 ▸). In addition, benzimidazole derivatives are an important class of chemicals with regard to their activity against several viruses such as HIV, herpes (HSV-1), influenza, Epstein-Barr and Burkitt’s lymphoma (Ramla *et al.*, 2007 ▸), and their use as anti-cancer agents (Ottanà *et al.*, 2005 ▸).

5-Sulfosalicylic acid is a particularly strong organic acid, which is capable of protonating N-containing heterocycles and other Lewis bases (Mu­thiah *et al.*, 2003 ▸) and thus can form structures with a variety of supra­molecular arrangements.
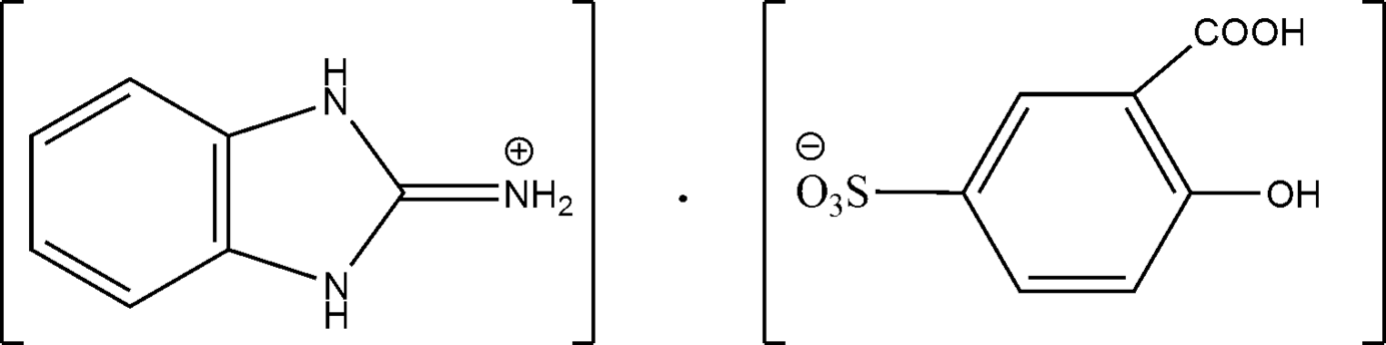


The present work was undertaken as part of our research program aimed at further understanding hydrogen-bonding inter­actions involving 2-amino­benzimidazole and 5-sulfo­salicylic acid. Here, we report the synthesis, crystal structure, and Hirshfeld surface analysis of the new organic salt 1,3-di­hydro-2*H*-benzimidazol-2-iminium 2-hy­droxy-5-sulfobenzoate, C_7_H_8_N_3_^+^·C_7_H_5_O_6_S^−^.

## 2. Structural commentary

**Figure 1:**
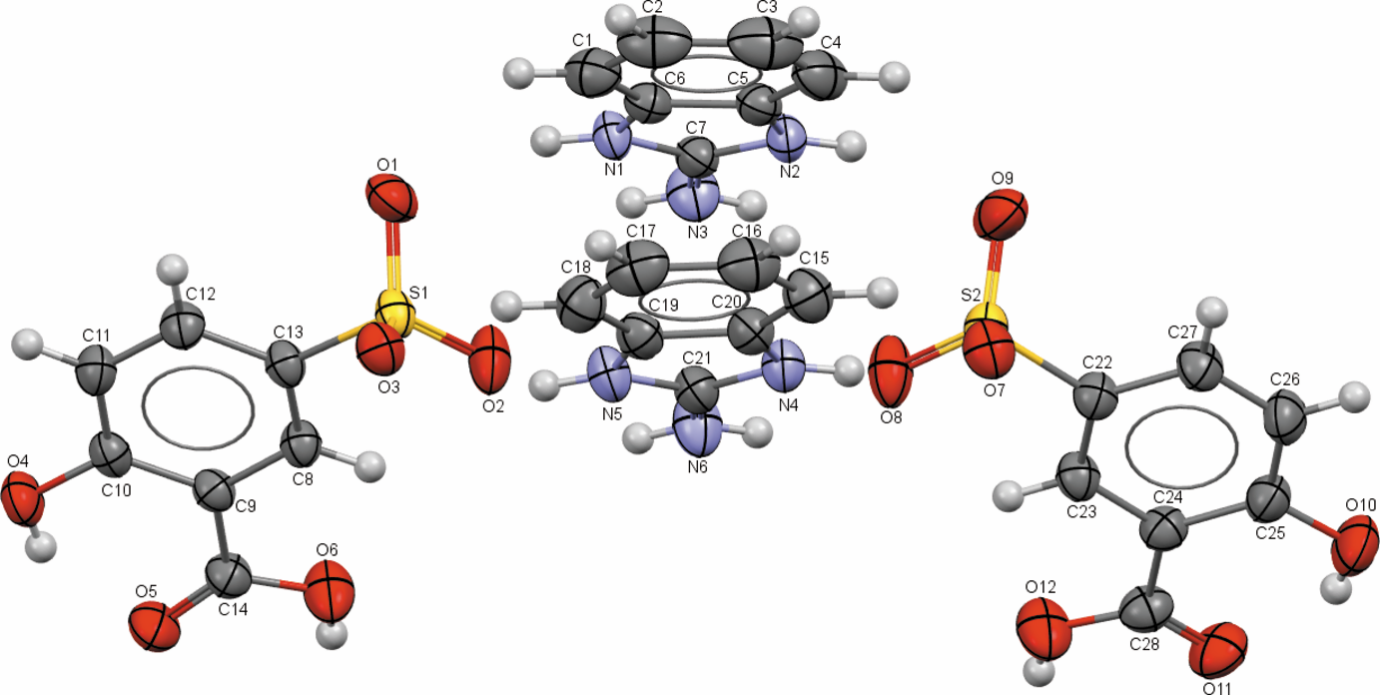
The structures of the mol­ecular entities in the title salt. Displacement ellipsoids are drawn at the 50% probability level.

The asymmetric unit of the title salt (Fig. 1 ▸) contains two 1,3-di­hydro-2*H*-benzimidazol-2-iminium cations and two 2-hy­droxy-5-sulfobenzoate anions (*Z*′ = 2). The N—C and S—O bond lengths range from 1.318 (2) to 1.394 (2) Å and from 1.4417 (12) to 1.4727 (12) Å, respectively. The O—S—O and N—C—N angles range from 110.60 (7) to 113.71 (8)° and from 109.10 (13) to 125.67 (15)°, respectively (Table 1 ▸). Overlays of the two cations and the two anions show that they are almost identical (Figs. S1 and S2 in the ESI), with somewhat greater deviations between the two anions. Analysis of bond lengths and angles shows that these data differ only slightly from those of other related compounds with similar structural units (Saiadali Fathima *et al.*, 2019 ▸; Atria *et al.*, 2012 ▸; Low *et al.*, 2003 ▸; ESI Table S1).

**Figure 2:**
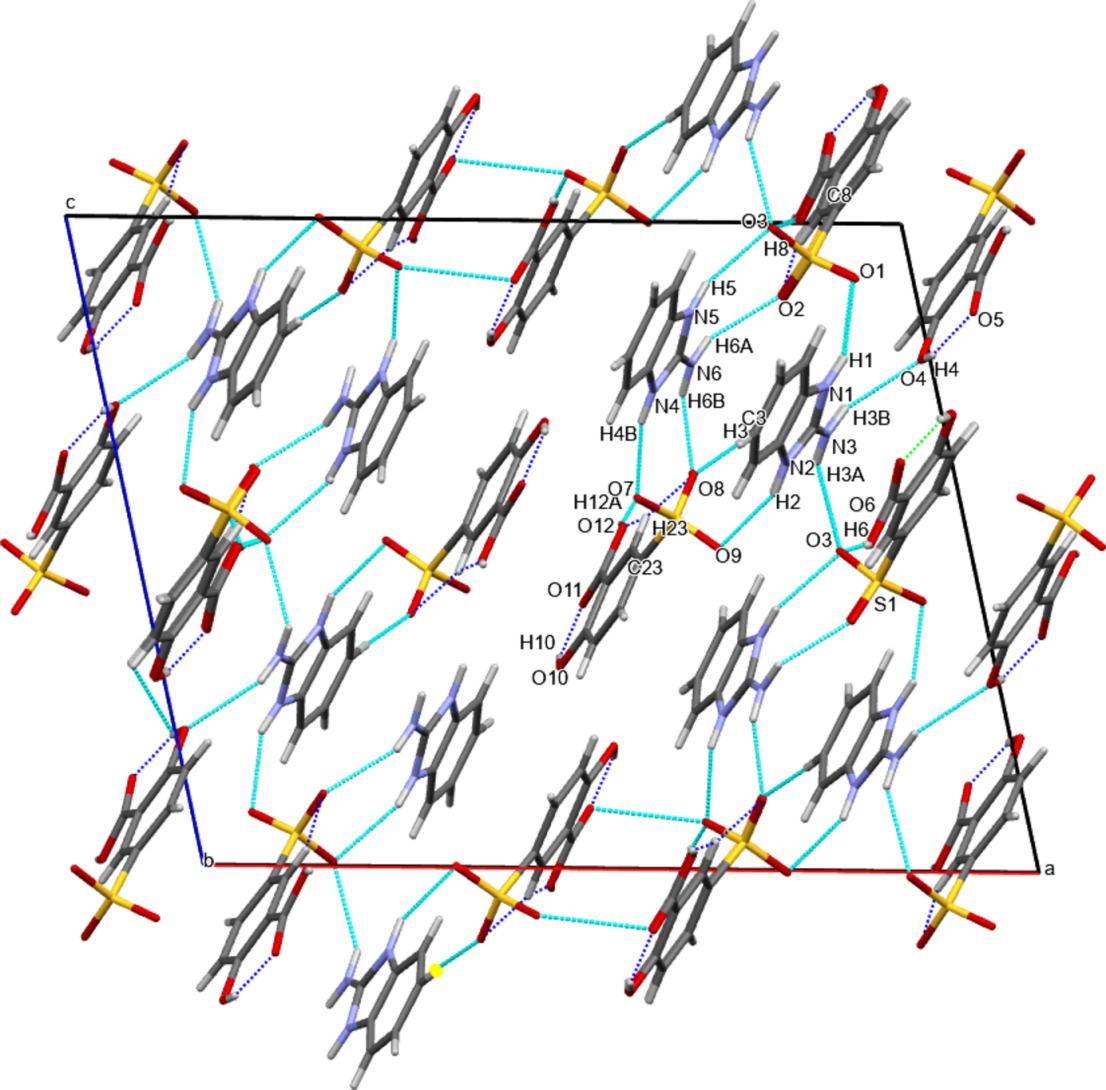
Crystal packing in a view along the *b* axis. Inter­molecular N—H⋯O and O—H⋯O inter­actions are shown as light-blue dashed lines and intra­molecular O—H⋯O inter­actions as dark-blue dashed lines.

An intra­molecular O—H⋯O hydrogen bond between the hy­droxy group and the non-protonated O atom of the carb­oxy group stabilizes the mol­ecular conformation for each of the cations (O4—H4⋯O5; O10—H10⋯O11; Fig. 2 ▸, Table 2 ▸).

## 3. Supra­molecular features

**Figure 3:**
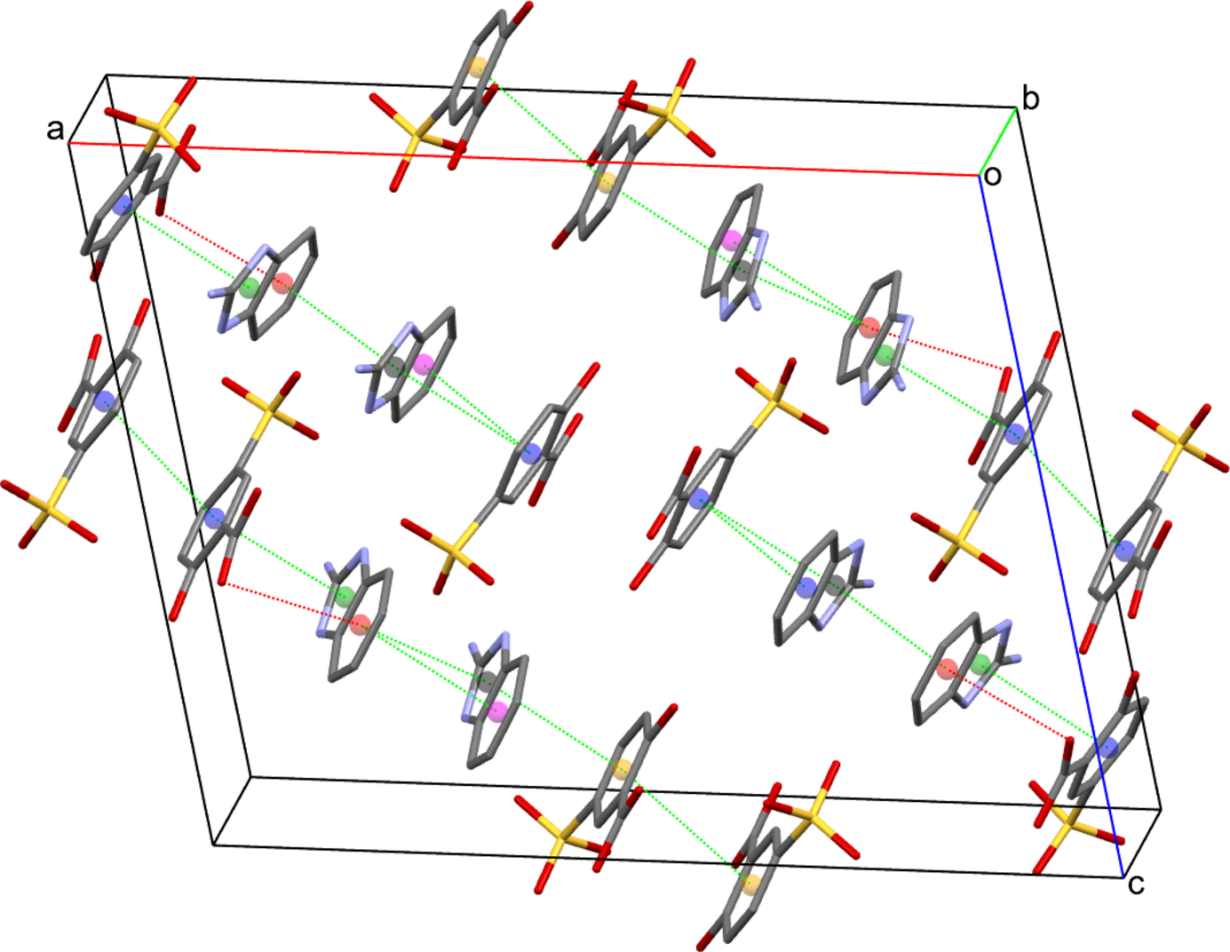
Inter­actions between aromatic rings in the title salt, leading to π–π stacking between the following ring centroids (*Cg*) shown as colored spheres: *Cg*1 (C8–C13, purple sphere); *Cg*2 (C22–C27, yellow sphere); *Cg*3 (N1/C5–C7/N2, green sphere); *Cg*4 (C1–C6, red sphere); *Cg*6 (N4/C19–C21/N5, black sphere); *Cg*7 (C15–C20, magenta sphere). The dashed red lines represent C14=O5⋯*Cg*4 contacts [3.7089 (19) Å].

In the crystal, the N atoms of the imidazolium cation form inter­molecular N—H⋯O hydrogen bonds with oxygen atoms of the sulfate group of the two hy­droxy­benzoate anions. Moreover, O—H⋯O inter­actions between the carb­oxy group and one of the sulfonate O atoms are present (Fig. 2 ▸, Table 2 ▸). Additionally, π–π inter­actions between the aromatic rings with centroid-to-centroid distances between 3.5094 (9) and 3.9824 (10) Å (numerical details are given in ESI Table S2) as well as C14=O5⋯*Cg*4(*x*, 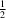 − *y*, −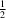 + *z*) inter­actions of 3.7089 (19) Å are present (Fig. 3 ▸). All of the above contacts contribute to the tri-periodic packing of the mol­ecular entities in the crystal.

## 4. Hirshfeld surface analysis

**Figure 4:**
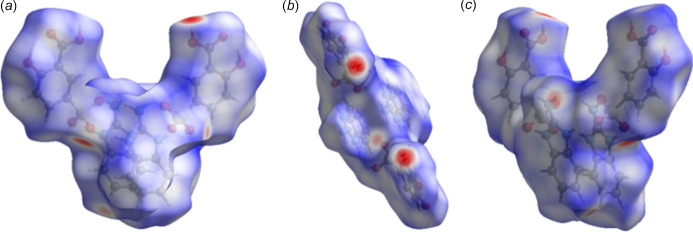
HS plotted over *d*_norm_ (*a*) along the *a* axis, (*b*) along the *b* axis and (*c*) along the *c* axis.

**Figure 5:**
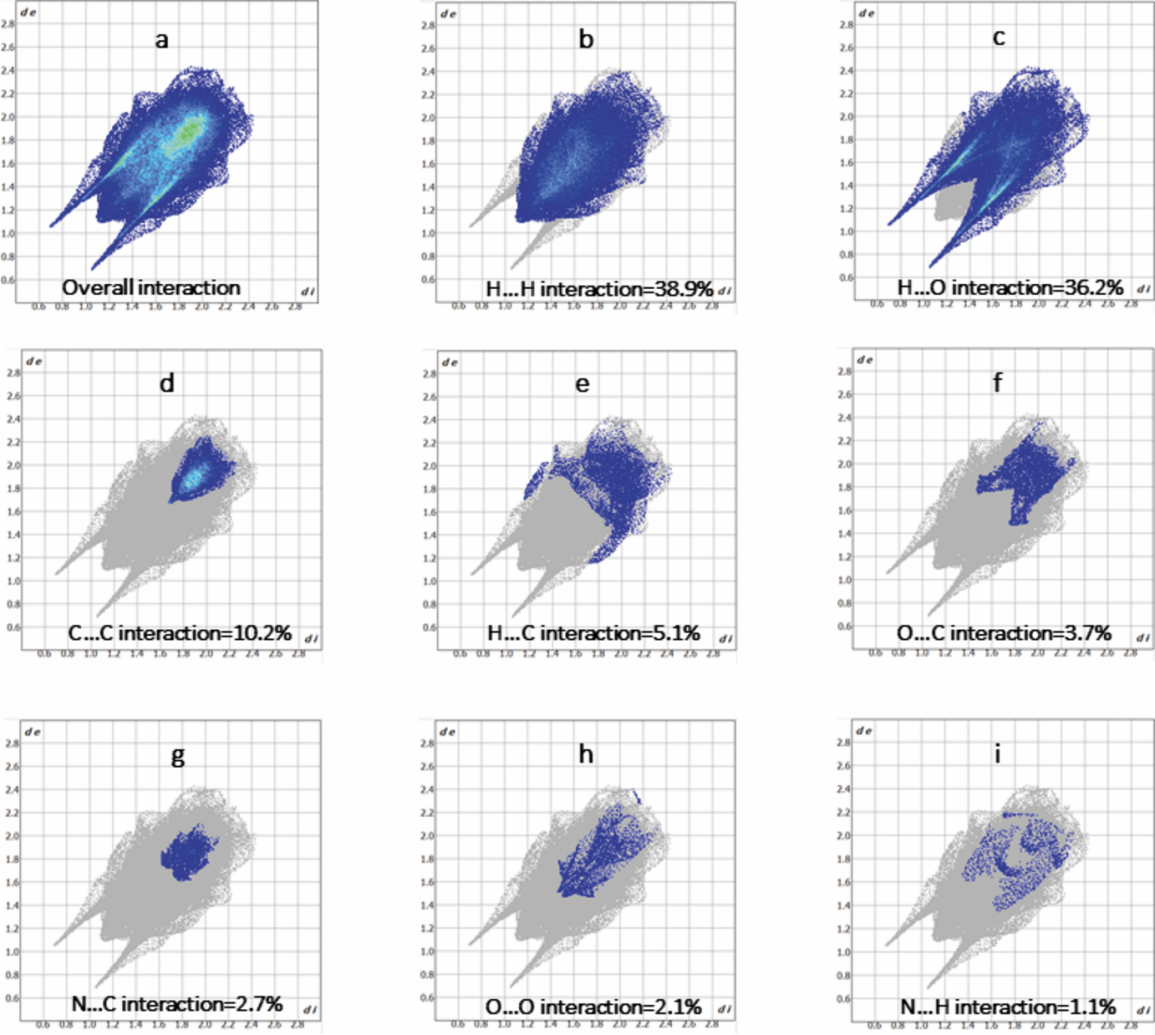
Two-dimensional fingerprint plots for (*a*) all inter­actions and (*b*)–(*i*) individual inter­atomic contacts.

In order to qu­antify the inter­molecular inter­actions in the title salt, the Hirshfeld surface (HS) (Spackman & Jayatilaka, 2009 ▸) was analysed and the associated two-dimensional fingerprint plots (McKinnon *et al.*, 2007 ▸) calculated with *CrystalExplorer* (Spackman *et al.*, 2021 ▸). The HS mapped over *d*_norm_ is represented in Fig. 4 ▸. White surface areas indicate contacts with distances equal to the sum of van der Waals radii, whereas red and blue colors denote distances shorter or longer than the sum of the van der Waals radii. The red spots clearly visible in Fig. 4 ▸ emphasize the importance of classical hydrogen-bonding inter­actions in the title salt. The two-dimensional fingerprint plot for all contacts is depicted in Fig. 5 ▸*a*. H⋯H contacts are responsible for the largest contribution (38.9%) to the Hirshfeld surface (Fig. 5 ▸*b*). Besides these contacts, H⋯O/O⋯H (36.2%), C⋯C (10.2%), H⋯C/C⋯H (5.1%) and O⋯C/C⋯O (3.7%) inter­actions contribute significantly to the total Hirshfeld surface; their decomposed fingerprint plots are shown in Fig. 5 ▸*c*–*f*. The contributions of further contacts are only minor and amount to N⋯C/C⋯N (2.6%), O⋯O (2.1%) and N⋯H/H⋯N (1.1%).

## 5. Database survey

A survey of the Cambridge Structural Database (CSD, version 5.43, update of November 2022; Groom *et al.*, 2016 ▸) revealed 47 hits related to the 2-amino­benzimidazolium cation. Among them are those that form inter­molecular hydrogen bonds like in the title salt: 2-amino­benzimidazolium hydrogen sulfate (DOKZEJ: You *et al.*, 2009 ▸), 2-amino­benzimidazolium *O*-ethyl malonate (EMIHAJ: Low *et al.*, 2003 ▸), 2-amino-1*H*-benzimidazol-3-ium 1,3-dioxo-1,3-di­hydro-2*H*-isoindol-2-olate 2-hy­droxy-1*H*-iso­indole-1,3(2*H*)-dione (EPETOK: Mahendiran *et al.*, 2016 ▸), 2-amino­benzimidazolium picrate (HUZSIE: El-Medani *et al.*, 2003 ▸), 2-amino-1*H*-benzimidazol-3-ium 2-propanamido­benzoat (NUVZIQ: Amor *et al.*, 2020 ▸) and 2-amino-1*H*-benzimidazol-3-ium pyridine-3-carboxyl­ate (VARCOJ: Fathima *et al.*, 2017 ▸). Eight hits containing sulfosalicylic acid in related organic salts were identified, among them 5-sulfosalicylic acid thio­urea (ETABAC: Xiong *et al.*, 2003 ▸), 2-hy­droxy-5-sulfo­benzoic acid aniline monohydrate (JUCJOG: Bakasova *et al.*, 1991 ▸), tris­(benzohydrazido)­cobalt chloride hydroxide 2-hy­droxy-5-sulfo­benzoic acid monohydrate (MOWTAV: Antsyshkina *et al.*, 2014 ▸). In all these structures inter­molecular hydrogen bonds are the dominant motif in the crystal packing.

## 6. Synthesis and crystallization

All reagents for synthesis and analysis were commercially available and purchased from Sigma Aldrich and used as received without further purification.

Sulfosalicylic acid and 2-amino­benzimidazole were reacted in a molar ratio of 1:1, using a solution of 0.133 g of 2-amino­benzimidazole in 5 ml of ethanol that was added dropwise to 0.228 g of the acid dissolved in 5 ml of ethanol. The mixture was stirred on a magnetic stirrer for 4 h. During the reaction time, the color of the solution changed from transparent to light brown. The solution was left for 2 weeks at room temperature for crystal growth. The formed crystals were filtered off and washed several times with ethanol to remove impurities.

## 7. Refinement

Crystal data, data collection and structure refinement details are summarized in Table 3 ▸. All H atoms were geometrically placed with C—H = 0.93 Å, O—H = 0.82 Å, N—H = 0.86 Å and with *U*_iso_(H) = 1.2*U*_eq_(C) and *U*_iso_(H) = 1.5*U_e_*_q_(O,N).

## Supplementary Material

Crystal structure: contains datablock(s) I. DOI: 10.1107/S2056989024008557/wm5724sup1.cif

Structure factors: contains datablock(s) I. DOI: 10.1107/S2056989024008557/wm5724Isup3.hkl

Table S1. DOI: 10.1107/S2056989024008557/wm5724sup4.docx

Table S2. DOI: 10.1107/S2056989024008557/wm5724sup5.docx

Fig. S1. DOI: 10.1107/S2056989024008557/wm5724sup6.tif

Fig. S2. DOI: 10.1107/S2056989024008557/wm5724sup7.tif

Supporting information file. DOI: 10.1107/S2056989024008557/wm5724Isup7.cml

CCDC reference: 2380553

Additional supporting information:  crystallographic information; 3D view; checkCIF report

## Figures and Tables

**Table 1:** Selected geometric parameters (Å, °)

S1—O3	1.4727 (12)	N1—C6	1.3886 (19)
S1—O1	1.4479 (13)	N2—C7	1.3350 (19)
S1—O2	1.4417 (12)	N2—C5	1.394 (2)
S1—C13	1.7634 (15)	N4—C21	1.340 (2)
S2—O7	1.4615 (12)	N4—C20	1.391 (2)
S2—O8	1.4455 (12)	N5—C21	1.335 (2)
S2—O9	1.4516 (14)	N5—C19	1.391 (2)
S2—C22	1.7645 (16)	N3—C7	1.322 (2)
N1—C7	1.3390 (19)	N6—C21	1.318 (2)
			
O1—S1—O3	110.60 (7)	N2—C7—N1	109.10 (13)
O2—S1—O3	111.98 (8)	N3—C7—N1	125.51 (14)
O2—S1—O1	113.71 (8)	N3—C7—N2	125.39 (15)
O8—S2—O7	111.30 (8)	N5—C21—N4	108.91 (14)
O8—S2—O9	113.02 (9)	N6—C21—N4	125.42 (15)
O9—S2—O7	111.40 (8)	N6—C21—N5	125.67 (15)

**Table 2:** Hydrogen-bond geometry (Å, °)

*D*—H⋯*A*	*D*—H	H⋯*A*	*D*⋯*A*	*D*—H⋯*A*
N1—H1⋯O1	0.86	2.08	2.8645 (16)	152
N2—H2⋯O9	0.86	2.07	2.8799 (19)	157
N3—H3*A*⋯O3^i^	0.86	2.37	3.0153 (19)	132
N3—H3*B*⋯O4^ii^	0.86	2.32	3.005 (2)	137
N4—H4*B*⋯O7	0.86	2.04	2.8760 (17)	163
N5—H5⋯O3	0.86	2.22	3.0331 (18)	158
N6—H6*A*⋯O2	0.86	2.17	2.877 (2)	140
N6—H6*B*⋯O8	0.86	2.06	2.845 (2)	152
O4—H4⋯O5	0.82	1.88	2.6007 (17)	147
O6—H6⋯O3^iii^	0.82	1.89	2.6958 (16)	165
O10—H10⋯O11	0.82	1.90	2.619 (2)	146
O12—H12*A*⋯O7^iii^	0.82	1.89	2.6746 (17)	159

Symmetry codes: (i) 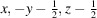; (ii) 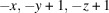; (iii) 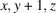.

**Table 3:** Experimental details

Crystal data	
Chemical formula	C_7_H_8_N_3_^+^·C_7_H_5_O_6_S^−^
*M*_r_	351.33
Crystal system, space group	Monoclinic, *P*2_1_/*c*
Temperature (K)	293
*a*, *b*, *c* (Å)	21.03214 (18), 8.61349 (6), 16.69445 (13)
β (°)	102.5549 (8)
*V* (Å^3^)	2952.05 (4)
*Z*	8
Radiation type	Cu *K*α
μ (mm^−1^)	2.33
Crystal size (mm)	0.48 × 0.16 × 0.08

Data collection	
Diffractometer	XtaLAB Synergy, Single source at home/near, HyPix3000
Absorption correction	Multi-scan (*CrysAlis PRO*; Rigaku OD, 2020 ▸)
*T*_min_, *T*_max_	0.871, 1.000
No. of measured, independent and observed [*I* > 2σ(*I*)] reflections	29960, 5715, 5051
*R*_int_	0.029
(sin θ/λ)_max_ (Å^−1^)	0.615

Refinement	
*R*[*F*^2^ > 2σ(*F*^2^)], *wR*(*F*^2^), *S*	0.034, 0.099, 1.06
No. of reflections	5715
No. of parameters	438
H-atom treatment	H-atom parameters constrained
Δρ_max_, Δρ_min_ (e Å^−3^)	0.27, −0.36

Computer programs: *CrysAlis PRO* (Rigaku OD, 2020 ▸), *SHELXT* (Sheldrick, 2015*a* ▸), *SHELXL* (Sheldrick, 2015*b* ▸) and *OLEX2* (Dolomanov *et al.*, 2009 ▸).

## supplementary crystallographic information

### 1,3-Dihydro-2H-benzimidazol-2-iminium 3-carboxy-4-hydroxybenzenesulfonate Crystal data

**Table:**

C_7_H_8_N_3_^+^·C_7_H_5_O_6_S^−^	*F*(000) = 1456
*M**_r_* = 351.33	*D*_x_ = 1.581 Mg m^−^^3^
Monoclinic, *P*2_1_/*c*	Cu *K*α radiation, λ = 1.54184 Å
*a* = 21.03214 (18) Å	Cell parameters from 16513 reflections
*b* = 8.61349 (6) Å	θ = 2.7–71.3°
*c* = 16.69445 (13) Å	µ = 2.33 mm^−^^1^
β = 102.5549 (8)°	*T* = 293 K
*V* = 2952.05 (4) Å^3^	Block, light brown
*Z* = 8	0.48 × 0.16 × 0.08 mm

### 1,3-Dihydro-2H-benzimidazol-2-iminium 3-carboxy-4-hydroxybenzenesulfonate Data collection

**Table:**

XtaLAB Synergy, Single source at home/near, HyPix3000 diffractometer	5715 independent reflections
Radiation source: micro-focus sealed X-ray tube, PhotonJet (Cu) X-ray Source	5051 reflections with *I* > 2σ(*I*)
Mirror monochromator	*R*_int_ = 0.029
Detector resolution: 10.0000 pixels mm^-1^	θ_max_ = 71.5°, θ_min_ = 4.3°
ω scans	*h* = −25→25
Absorption correction: multi-scan (CrysAlis PRO; Rigaku OD, 2020)	*k* = −10→10
*T*_min_ = 0.871, *T*_max_ = 1.000	*l* = −20→20
29960 measured reflections	

### 1,3-Dihydro-2H-benzimidazol-2-iminium 3-carboxy-4-hydroxybenzenesulfonate Refinement

**Table:**

Refinement on *F*^2^	Hydrogen site location: inferred from neighbouring sites
Least-squares matrix: full	H-atom parameters constrained
*R*[*F*^2^ > 2σ(*F*^2^)] = 0.034	*w* = 1/[σ^2^(*F*_o_^2^) + (0.0567*P*)^2^ + 0.6854*P*] where *P* = (*F*_o_^2^ + 2*F*_c_^2^)/3
*wR*(*F*^2^) = 0.099	(Δ/σ)_max_ = 0.001
*S* = 1.06	Δρ_max_ = 0.27 e Å^−^^3^
5715 reflections	Δρ_min_ = −0.36 e Å^−^^3^
438 parameters	Extinction correction: SHELXL2016/6 (Sheldrick, 2015b), Fc^*^=kFc[1+0.001xFc^2^λ^3^/sin(2θ)]^-1/4^
0 restraints	Extinction coefficient: 0.00055 (7)
Primary atom site location: dual	

### 1,3-Dihydro-2H-benzimidazol-2-iminium 3-carboxy-4-hydroxybenzenesulfonate Special details

**Table:**

Geometry. All esds (except the esd in the dihedral angle between two l.s. planes) are estimated using the full covariance matrix. The cell esds are taken into account individually in the estimation of esds in distances, angles and torsion angles; correlations between esds in cell parameters are only used when they are defined by crystal symmetry. An approximate (isotropic) treatment of cell esds is used for estimating esds involving l.s. planes.

### 1,3-Dihydro-2H-benzimidazol-2-iminium 3-carboxy-4-hydroxybenzenesulfonate Fractional atomic coordinates and isotropic or equivalent isotropic displacement parameters (Å^2^)

**Table:**

	*x*	*y*	*z*	*U*_iso_*/*U*_eq_	
S1	0.11934 (2)	0.24229 (4)	0.55062 (2)	0.03300 (11)	
S2	0.34651 (2)	0.27387 (4)	0.95601 (2)	0.03508 (12)	
O3	0.15655 (6)	0.14053 (13)	0.50668 (7)	0.0400 (3)	
O4	−0.01170 (6)	0.66757 (14)	0.29142 (7)	0.0440 (3)	
H4	0.003130	0.755665	0.299245	0.066*	
O1	0.07143 (6)	0.15365 (15)	0.58177 (7)	0.0470 (3)	
O7	0.38626 (6)	0.15583 (13)	0.92733 (7)	0.0437 (3)	
O5	0.06294 (6)	0.88704 (14)	0.36682 (7)	0.0471 (3)	
O6	0.12027 (7)	0.83981 (13)	0.49325 (8)	0.0538 (4)	
H6	0.124383	0.934506	0.494093	0.081*	
O2	0.16137 (7)	0.33480 (14)	0.61221 (7)	0.0533 (4)	
O8	0.31346 (7)	0.37100 (14)	0.88921 (8)	0.0548 (4)	
O12	0.41101 (8)	0.86010 (14)	0.97231 (8)	0.0570 (4)	
H12A	0.411808	0.954869	0.967980	0.085*	
O11	0.47871 (7)	0.91026 (15)	1.09195 (9)	0.0538 (3)	
O9	0.30261 (6)	0.20503 (17)	1.00215 (8)	0.0545 (3)	
O10	0.52275 (7)	0.67731 (17)	1.18904 (8)	0.0574 (4)	
H10	0.519921	0.768036	1.173804	0.086*	
N1	0.13245 (6)	0.09746 (15)	0.75004 (7)	0.0341 (3)	
H1	0.103915	0.126568	0.707628	0.041*	
N2	0.19964 (6)	0.10264 (16)	0.86938 (8)	0.0356 (3)	
H2	0.221336	0.135381	0.916026	0.043*	
N4	0.33971 (7)	0.05422 (16)	0.76079 (8)	0.0377 (3)	
H4B	0.361431	0.081968	0.808392	0.045*	
N5	0.27558 (7)	0.06439 (16)	0.63948 (8)	0.0399 (3)	
H5	0.249491	0.099795	0.596411	0.048*	
N3	0.14323 (8)	0.33421 (16)	0.82363 (9)	0.0463 (4)	
H3A	0.161926	0.384146	0.866979	0.056*	
H3B	0.115343	0.380427	0.785840	0.056*	
N6	0.28709 (8)	0.29260 (18)	0.71966 (10)	0.0509 (4)	
H6A	0.261023	0.344077	0.682173	0.061*	
H6B	0.304792	0.336881	0.765226	0.061*	
C8	0.09424 (7)	0.52774 (17)	0.48123 (9)	0.0314 (3)	
H8	0.125957	0.563268	0.525213	0.038*	
C9	0.06462 (7)	0.63126 (17)	0.42016 (9)	0.0303 (3)	
C7	0.15735 (8)	0.18636 (18)	0.81509 (9)	0.0327 (3)	
C6	0.16017 (7)	−0.04933 (18)	0.76213 (9)	0.0327 (3)	
C10	0.01692 (7)	0.57477 (18)	0.35440 (9)	0.0319 (3)	
C5	0.20288 (7)	−0.04648 (18)	0.83812 (9)	0.0341 (3)	
C13	0.07684 (7)	0.37327 (17)	0.47686 (9)	0.0308 (3)	
C22	0.40035 (7)	0.39564 (18)	1.02406 (9)	0.0338 (3)	
C12	0.02746 (8)	0.31920 (18)	0.41300 (9)	0.0352 (3)	
H12	0.014833	0.215563	0.411341	0.042*	
C14	0.08204 (8)	0.79738 (18)	0.42341 (10)	0.0360 (3)	
C21	0.29989 (8)	0.14585 (19)	0.70729 (9)	0.0362 (3)	
C11	−0.00241 (8)	0.42021 (18)	0.35255 (9)	0.0351 (3)	
H11	−0.035660	0.384926	0.310287	0.042*	
C19	0.29916 (8)	−0.0868 (2)	0.64961 (10)	0.0366 (3)	
C25	0.48227 (8)	0.5887 (2)	1.13349 (10)	0.0390 (4)	
C23	0.40528 (8)	0.55034 (18)	1.00533 (9)	0.0348 (3)	
H23	0.381287	0.589129	0.955959	0.042*	
C27	0.43664 (8)	0.33542 (19)	1.09781 (10)	0.0400 (4)	
H27	0.433598	0.230805	1.110242	0.048*	
C20	0.34016 (8)	−0.09308 (19)	0.72674 (10)	0.0361 (3)	
C26	0.47681 (8)	0.4322 (2)	1.15167 (11)	0.0442 (4)	
H26	0.500674	0.392696	1.200933	0.053*	
C24	0.44597 (8)	0.64935 (18)	1.05978 (10)	0.0348 (3)	
C1	0.15138 (9)	−0.1813 (2)	0.71355 (11)	0.0458 (4)	
H1A	0.123446	−0.182244	0.662054	0.055*	
C28	0.44777 (8)	0.81802 (19)	1.04362 (11)	0.0409 (4)	
C15	0.37191 (9)	−0.2290 (2)	0.75666 (12)	0.0452 (4)	
H15	0.399729	−0.233354	0.808185	0.054*	
C4	0.23850 (9)	−0.1770 (2)	0.86892 (12)	0.0500 (5)	
H4A	0.267711	−0.175518	0.919486	0.060*	
C18	0.28712 (9)	−0.2161 (2)	0.59962 (11)	0.0462 (4)	
H18	0.259158	−0.211927	0.548186	0.055*	
C16	0.36023 (9)	−0.3572 (2)	0.70630 (13)	0.0514 (5)	
H16	0.380914	−0.450282	0.724237	0.062*	
C17	0.31836 (10)	−0.3514 (2)	0.62939 (12)	0.0517 (5)	
H17	0.311335	−0.440819	0.597426	0.062*	
C2	0.18617 (11)	−0.3113 (2)	0.74543 (14)	0.0577 (5)	
H2A	0.180926	−0.402913	0.715202	0.069*	
C3	0.22868 (10)	−0.3085 (2)	0.82137 (15)	0.0599 (6)	
H3	0.251307	−0.398462	0.840797	0.072*	

### 1,3-Dihydro-2H-benzimidazol-2-iminium 3-carboxy-4-hydroxybenzenesulfonate Atomic displacement parameters (Å^2^)

**Table:**

	*U*^11^	*U*^22^	*U*^33^	*U*^12^	*U*^13^	*U*^23^
S1	0.0425 (2)	0.02530 (18)	0.02788 (19)	0.00298 (14)	0.00036 (15)	0.00239 (13)
S2	0.0397 (2)	0.02640 (19)	0.0354 (2)	0.00288 (15)	−0.00023 (16)	−0.00232 (14)
O3	0.0455 (6)	0.0324 (6)	0.0411 (6)	0.0087 (5)	0.0071 (5)	0.0030 (5)
O4	0.0517 (7)	0.0398 (6)	0.0348 (6)	0.0040 (5)	−0.0032 (5)	0.0100 (5)
O1	0.0532 (7)	0.0468 (7)	0.0415 (6)	0.0019 (6)	0.0117 (5)	0.0151 (5)
O7	0.0562 (7)	0.0301 (6)	0.0429 (6)	0.0088 (5)	0.0067 (5)	−0.0024 (5)
O5	0.0556 (7)	0.0336 (6)	0.0481 (7)	−0.0027 (5)	0.0025 (6)	0.0135 (5)
O6	0.0771 (9)	0.0260 (6)	0.0485 (7)	−0.0054 (6)	−0.0076 (6)	0.0012 (5)
O2	0.0699 (8)	0.0341 (6)	0.0420 (7)	0.0021 (6)	−0.0182 (6)	−0.0023 (5)
O8	0.0699 (9)	0.0343 (6)	0.0467 (7)	0.0102 (6)	−0.0168 (6)	−0.0016 (5)
O12	0.0818 (10)	0.0277 (6)	0.0537 (8)	−0.0024 (6)	−0.0021 (7)	0.0057 (6)
O11	0.0555 (8)	0.0354 (6)	0.0666 (8)	−0.0102 (6)	0.0047 (6)	−0.0057 (6)
O9	0.0484 (7)	0.0638 (8)	0.0509 (7)	−0.0183 (6)	0.0099 (6)	−0.0115 (7)
O10	0.0562 (8)	0.0524 (8)	0.0528 (8)	−0.0137 (7)	−0.0117 (6)	−0.0037 (6)
N1	0.0405 (7)	0.0321 (7)	0.0262 (6)	0.0022 (5)	0.0000 (5)	0.0030 (5)
N2	0.0375 (7)	0.0386 (7)	0.0274 (6)	−0.0016 (6)	0.0001 (5)	0.0018 (5)
N4	0.0409 (7)	0.0364 (7)	0.0328 (7)	0.0032 (6)	0.0011 (5)	−0.0011 (5)
N5	0.0474 (8)	0.0405 (7)	0.0293 (7)	0.0064 (6)	0.0026 (6)	0.0017 (6)
N3	0.0593 (9)	0.0318 (7)	0.0452 (8)	0.0039 (7)	0.0056 (7)	−0.0024 (6)
N6	0.0648 (10)	0.0365 (8)	0.0449 (8)	0.0093 (7)	−0.0024 (7)	−0.0017 (6)
C8	0.0353 (8)	0.0284 (7)	0.0289 (7)	0.0021 (6)	0.0031 (6)	−0.0002 (6)
C9	0.0335 (7)	0.0274 (7)	0.0300 (7)	0.0025 (6)	0.0070 (6)	0.0021 (6)
C7	0.0365 (8)	0.0321 (8)	0.0300 (7)	−0.0017 (6)	0.0078 (6)	0.0024 (6)
C6	0.0350 (8)	0.0301 (7)	0.0342 (8)	0.0000 (6)	0.0101 (6)	0.0037 (6)
C10	0.0336 (8)	0.0348 (8)	0.0272 (7)	0.0063 (6)	0.0064 (6)	0.0031 (6)
C5	0.0330 (8)	0.0339 (8)	0.0361 (8)	0.0001 (6)	0.0090 (6)	0.0070 (6)
C13	0.0364 (8)	0.0280 (7)	0.0270 (7)	0.0039 (6)	0.0047 (6)	0.0007 (6)
C22	0.0351 (8)	0.0285 (7)	0.0362 (8)	0.0029 (6)	0.0041 (6)	−0.0002 (6)
C12	0.0406 (8)	0.0285 (7)	0.0343 (8)	−0.0007 (6)	0.0032 (6)	−0.0009 (6)
C14	0.0386 (8)	0.0305 (8)	0.0378 (8)	0.0005 (7)	0.0056 (6)	0.0038 (7)
C21	0.0395 (8)	0.0364 (8)	0.0323 (8)	0.0013 (7)	0.0069 (6)	0.0030 (6)
C11	0.0362 (8)	0.0358 (8)	0.0302 (7)	0.0002 (6)	0.0007 (6)	−0.0032 (6)
C19	0.0375 (8)	0.0387 (8)	0.0348 (8)	0.0023 (7)	0.0106 (6)	0.0008 (7)
C25	0.0349 (8)	0.0397 (9)	0.0398 (8)	−0.0035 (7)	0.0025 (7)	−0.0023 (7)
C23	0.0388 (8)	0.0299 (8)	0.0337 (8)	0.0039 (6)	0.0033 (6)	0.0017 (6)
C27	0.0429 (9)	0.0320 (8)	0.0415 (9)	0.0024 (7)	0.0012 (7)	0.0064 (7)
C20	0.0371 (8)	0.0365 (8)	0.0350 (8)	0.0017 (7)	0.0088 (6)	0.0013 (7)
C26	0.0431 (9)	0.0443 (9)	0.0390 (9)	0.0021 (8)	−0.0045 (7)	0.0071 (7)
C24	0.0353 (8)	0.0293 (8)	0.0394 (8)	0.0002 (6)	0.0070 (6)	0.0000 (6)
C1	0.0544 (10)	0.0393 (9)	0.0467 (10)	−0.0075 (8)	0.0176 (8)	−0.0069 (8)
C28	0.0420 (9)	0.0319 (8)	0.0492 (10)	−0.0023 (7)	0.0111 (7)	−0.0017 (7)
C15	0.0460 (10)	0.0425 (9)	0.0457 (10)	0.0068 (8)	0.0068 (8)	0.0054 (8)
C4	0.0416 (9)	0.0505 (11)	0.0578 (11)	0.0088 (8)	0.0105 (8)	0.0227 (9)
C18	0.0479 (10)	0.0491 (10)	0.0413 (9)	−0.0004 (8)	0.0089 (8)	−0.0094 (8)
C16	0.0510 (11)	0.0386 (9)	0.0665 (12)	0.0063 (8)	0.0167 (9)	0.0042 (9)
C17	0.0553 (11)	0.0408 (10)	0.0621 (12)	−0.0027 (8)	0.0196 (9)	−0.0131 (9)
C2	0.0669 (13)	0.0334 (9)	0.0813 (15)	−0.0004 (9)	0.0345 (11)	−0.0049 (9)
C3	0.0591 (12)	0.0354 (10)	0.0924 (16)	0.0144 (9)	0.0324 (12)	0.0181 (10)

### 1,3-Dihydro-2H-benzimidazol-2-iminium 3-carboxy-4-hydroxybenzenesulfonate Geometric parameters (Å, º)

**Table:**

S1—O3	1.4727 (12)	C8—C13	1.378 (2)
S1—O1	1.4479 (13)	C9—C10	1.403 (2)
S1—O2	1.4417 (12)	C9—C14	1.475 (2)
S1—C13	1.7634 (15)	C6—C5	1.386 (2)
S2—O7	1.4615 (12)	C6—C1	1.385 (2)
S2—O8	1.4455 (12)	C10—C11	1.390 (2)
S2—O9	1.4516 (14)	C5—C4	1.386 (2)
S2—C22	1.7645 (16)	C13—C12	1.397 (2)
O4—H4	0.8200	C22—C23	1.378 (2)
O4—C10	1.3533 (17)	C22—C27	1.400 (2)
O5—C14	1.2188 (19)	C12—H12	0.9300
O6—H6	0.8200	C12—C11	1.376 (2)
O6—C14	1.3160 (19)	C11—H11	0.9300
O12—H12A	0.8200	C19—C20	1.386 (2)
O12—C28	1.321 (2)	C19—C18	1.382 (2)
O11—C28	1.216 (2)	C25—C26	1.391 (2)
O10—H10	0.8200	C25—C24	1.401 (2)
O10—C25	1.3507 (19)	C23—H23	0.9300
N1—H1	0.8600	C23—C24	1.396 (2)
N1—C7	1.3390 (19)	C27—H27	0.9300
N1—C6	1.3886 (19)	C27—C26	1.374 (2)
N2—H2	0.8600	C20—C15	1.386 (2)
N2—C7	1.3350 (19)	C26—H26	0.9300
N2—C5	1.394 (2)	C24—C28	1.480 (2)
N4—H4B	0.8600	C1—H1A	0.9300
N4—C21	1.340 (2)	C1—C2	1.380 (3)
N4—C20	1.391 (2)	C15—H15	0.9300
N5—H5	0.8600	C15—C16	1.378 (3)
N5—C21	1.335 (2)	C4—H4A	0.9300
N5—C19	1.391 (2)	C4—C3	1.373 (3)
N3—H3A	0.8600	C18—H18	0.9300
N3—H3B	0.8600	C18—C17	1.377 (3)
N3—C7	1.322 (2)	C16—H16	0.9300
N6—H6A	0.8600	C16—C17	1.391 (3)
N6—H6B	0.8600	C17—H17	0.9300
N6—C21	1.318 (2)	C2—H2A	0.9300
C8—H8	0.9300	C2—C3	1.384 (3)
C8—C9	1.395 (2)	C3—H3	0.9300
			
O3—S1—C13	106.05 (7)	C11—C12—C13	119.60 (14)
O1—S1—O3	110.60 (7)	C11—C12—H12	120.2
O1—S1—C13	107.55 (7)	O5—C14—O6	123.07 (15)
O2—S1—O3	111.98 (8)	O5—C14—C9	123.28 (14)
O2—S1—O1	113.71 (8)	O6—C14—C9	113.65 (13)
O2—S1—C13	106.47 (7)	N5—C21—N4	108.91 (14)
O7—S2—C22	106.97 (7)	N6—C21—N4	125.42 (15)
O8—S2—O7	111.30 (8)	N6—C21—N5	125.67 (15)
O8—S2—O9	113.02 (9)	C10—C11—H11	119.9
O8—S2—C22	106.64 (7)	C12—C11—C10	120.29 (14)
O9—S2—O7	111.40 (8)	C12—C11—H11	119.9
O9—S2—C22	107.12 (8)	C20—C19—N5	106.42 (14)
C10—O4—H4	109.5	C18—C19—N5	131.89 (15)
C14—O6—H6	109.5	C18—C19—C20	121.67 (16)
C28—O12—H12A	109.5	O10—C25—C26	117.85 (15)
C25—O10—H10	109.5	O10—C25—C24	122.28 (15)
C7—N1—H1	125.5	C26—C25—C24	119.86 (15)
C7—N1—C6	108.96 (12)	C22—C23—H23	119.7
C6—N1—H1	125.5	C22—C23—C24	120.60 (14)
C7—N2—H2	125.6	C24—C23—H23	119.7
C7—N2—C5	108.86 (13)	C22—C27—H27	120.3
C5—N2—H2	125.6	C26—C27—C22	119.40 (15)
C21—N4—H4B	125.6	C26—C27—H27	120.3
C21—N4—C20	108.88 (13)	C19—C20—N4	106.61 (14)
C20—N4—H4B	125.6	C15—C20—N4	131.96 (15)
C21—N5—H5	125.4	C15—C20—C19	121.42 (16)
C21—N5—C19	109.16 (13)	C25—C26—H26	119.6
C19—N5—H5	125.4	C27—C26—C25	120.88 (15)
H3A—N3—H3B	120.0	C27—C26—H26	119.6
C7—N3—H3A	120.0	C25—C24—C28	119.73 (14)
C7—N3—H3B	120.0	C23—C24—C25	118.95 (14)
H6A—N6—H6B	120.0	C23—C24—C28	121.17 (14)
C21—N6—H6A	120.0	C6—C1—H1A	121.7
C21—N6—H6B	120.0	C2—C1—C6	116.63 (18)
C9—C8—H8	119.8	C2—C1—H1A	121.7
C13—C8—H8	119.8	O12—C28—C24	113.61 (14)
C13—C8—C9	120.48 (13)	O11—C28—O12	122.87 (16)
C8—C9—C10	118.67 (14)	O11—C28—C24	123.50 (16)
C8—C9—C14	121.67 (13)	C20—C15—H15	121.6
C10—C9—C14	119.66 (13)	C16—C15—C20	116.70 (17)
N2—C7—N1	109.10 (13)	C16—C15—H15	121.6
N3—C7—N1	125.51 (14)	C5—C4—H4A	121.5
N3—C7—N2	125.39 (15)	C3—C4—C5	116.97 (18)
C5—C6—N1	106.56 (13)	C3—C4—H4A	121.5
C1—C6—N1	131.59 (15)	C19—C18—H18	121.5
C1—C6—C5	121.85 (15)	C17—C18—C19	117.00 (17)
O4—C10—C9	121.64 (14)	C17—C18—H18	121.5
O4—C10—C11	118.02 (13)	C15—C16—H16	119.1
C11—C10—C9	120.34 (13)	C15—C16—C17	121.86 (18)
C6—C5—N2	106.52 (13)	C17—C16—H16	119.1
C6—C5—C4	121.03 (16)	C18—C17—C16	121.34 (17)
C4—C5—N2	132.45 (16)	C18—C17—H17	119.3
C8—C13—S1	119.50 (11)	C16—C17—H17	119.3
C8—C13—C12	120.49 (14)	C1—C2—H2A	119.3
C12—C13—S1	119.97 (12)	C1—C2—C3	121.47 (19)
C23—C22—S2	119.88 (12)	C3—C2—H2A	119.3
C23—C22—C27	120.30 (15)	C4—C3—C2	122.02 (18)
C27—C22—S2	119.80 (12)	C4—C3—H3	119.0
C13—C12—H12	120.2	C2—C3—H3	119.0
			
S1—C13—C12—C11	−175.55 (12)	C6—C1—C2—C3	−1.3 (3)
S2—C22—C23—C24	177.81 (12)	C10—C9—C14—O5	−9.1 (2)
S2—C22—C27—C26	−177.85 (14)	C10—C9—C14—O6	170.92 (15)
O3—S1—C13—C8	−111.11 (13)	C5—N2—C7—N1	0.55 (18)
O3—S1—C13—C12	66.71 (14)	C5—N2—C7—N3	−178.33 (15)
O4—C10—C11—C12	176.78 (14)	C5—C6—C1—C2	1.5 (3)
O1—S1—C13—C8	130.53 (13)	C5—C4—C3—C2	1.1 (3)
O1—S1—C13—C12	−51.65 (15)	C13—C8—C9—C10	0.0 (2)
O7—S2—C22—C23	117.00 (14)	C13—C8—C9—C14	179.72 (15)
O7—S2—C22—C27	−64.59 (15)	C13—C12—C11—C10	0.8 (2)
O2—S1—C13—C8	8.31 (15)	C22—C23—C24—C25	0.6 (2)
O2—S1—C13—C12	−173.88 (13)	C22—C23—C24—C28	−175.03 (16)
O8—S2—C22—C23	−2.19 (16)	C22—C27—C26—C25	−0.6 (3)
O8—S2—C22—C27	176.22 (14)	C14—C9—C10—O4	3.1 (2)
O9—S2—C22—C23	−123.46 (14)	C14—C9—C10—C11	−176.73 (14)
O9—S2—C22—C27	54.95 (15)	C21—N4—C20—C19	0.50 (18)
O10—C25—C26—C27	−179.84 (17)	C21—N4—C20—C15	−178.85 (18)
O10—C25—C24—C23	179.85 (16)	C21—N5—C19—C20	−1.00 (18)
O10—C25—C24—C28	−4.4 (2)	C21—N5—C19—C18	177.32 (18)
N1—C6—C5—N2	−0.16 (17)	C19—N5—C21—N4	1.34 (19)
N1—C6—C5—C4	179.65 (15)	C19—N5—C21—N6	−178.87 (17)
N1—C6—C1—C2	−178.53 (17)	C19—C20—C15—C16	−0.6 (3)
N2—C5—C4—C3	178.81 (17)	C19—C18—C17—C16	−0.1 (3)
N4—C20—C15—C16	178.71 (18)	C25—C24—C28—O12	−179.47 (16)
N5—C19—C20—N4	0.30 (18)	C25—C24—C28—O11	−1.2 (3)
N5—C19—C20—C15	179.73 (15)	C23—C22—C27—C26	0.6 (3)
N5—C19—C18—C17	−178.94 (18)	C23—C24—C28—O12	−3.8 (2)
C8—C9—C10—O4	−177.17 (14)	C23—C24—C28—O11	174.42 (17)
C8—C9—C10—C11	3.0 (2)	C27—C22—C23—C24	−0.6 (2)
C8—C9—C14—O5	171.10 (16)	C20—N4—C21—N5	−1.14 (19)
C8—C9—C14—O6	−8.8 (2)	C20—N4—C21—N6	179.06 (17)
C8—C13—C12—C11	2.2 (2)	C20—C19—C18—C17	−0.8 (3)
C9—C8—C13—S1	175.20 (11)	C20—C15—C16—C17	−0.4 (3)
C9—C8—C13—C12	−2.6 (2)	C26—C25—C24—C23	−0.7 (2)
C9—C10—C11—C12	−3.4 (2)	C26—C25—C24—C28	175.07 (17)
C7—N1—C6—C5	0.49 (17)	C24—C25—C26—C27	0.6 (3)
C7—N1—C6—C1	−179.48 (17)	C1—C6—C5—N2	179.82 (15)
C7—N2—C5—C6	−0.24 (17)	C1—C6—C5—C4	−0.4 (2)
C7—N2—C5—C4	179.98 (17)	C1—C2—C3—C4	0.0 (3)
C6—N1—C7—N2	−0.65 (17)	C15—C16—C17—C18	0.7 (3)
C6—N1—C7—N3	178.23 (16)	C18—C19—C20—N4	−178.23 (16)
C6—C5—C4—C3	−0.9 (3)	C18—C19—C20—C15	1.2 (3)

### 1,3-Dihydro-2H-benzimidazol-2-iminium 3-carboxy-4-hydroxybenzenesulfonate Hydrogen-bond geometry (Å, º)

**Table:**

*D*—H···*A*	*D*—H	H···*A*	*D*···*A*	*D*—H···*A*
N1—H1···O1	0.86	2.08	2.8645 (16)	152
N2—H2···O9	0.86	2.07	2.8799 (19)	157
N3—H3*A*···O3^i^	0.86	2.37	3.0153 (19)	132
N3—H3*B*···O4^ii^	0.86	2.32	3.005 (2)	137
N4—H4*B*···O7	0.86	2.04	2.8760 (17)	163
N5—H5···O3	0.86	2.22	3.0331 (18)	158
N6—H6*A*···O2	0.86	2.17	2.877 (2)	140
N6—H6*B*···O8	0.86	2.06	2.845 (2)	152
O4—H4···O5	0.82	1.88	2.6007 (17)	147
O6—H6···O3^iii^	0.82	1.89	2.6958 (16)	165
O10—H10···O11	0.82	1.90	2.619 (2)	146
O12—H12*A*···O7^iii^	0.82	1.89	2.6746 (17)	159
C3—H3···O8^iv^	0.93	2.42	3.347 (2)	178
C8—H8···O2	0.93	2.46	2.8626 (19)	106
C23—H23···O8	0.93	2.47	2.872 (2)	106
C23—H23···O12	0.93	2.42	2.732 (2)	100

Symmetry codes: (i) *x*, −*y*−1/2, *z*−1/2; (ii) −*x*, −*y*+1, −*z*+1; (iii) *x*, *y*+1, *z*; (iv) *x*, *y*−1, *z*.

## References

[bb1] Aboul-Enein, H. Y. & El Rashedy, A. A. (2015). *Med. Chem.* **5**, 318–325.

[bb2] Amor, A. B. H., Akriche, S., Arfaoui, Y. & Abderrahim, R. (2020). *J. Mol. Struct.* **1222**, 128921–128930.

[bb3] Antsyshkina, A. S., Koksharova, T. V., Sergienko, V. S., Mandzii, T. V. & Sadikov, G. G. (2014). *Russ. J. Inorg. Chem.* **59**, 1417–1423.

[bb4] Atria, A. M., Garland, M. T. & Baggio, R. (2012). *Acta Cryst.* C**68**, m185–m188. 10.1107/S0108270112024468 22763685

[bb5] Bakasova, Z. B., Abdybaliev, D. A., Sharipov, Kh. T., Akbaev, A. A., Ibragimov, R. T., Talipov, S. A. & Ismankulov, A. I. (1991). *Uzb. Khim. Zh.* pp. 22–25.

[bb6] Dokla, E. M. E., Abutaleb, N. S., Milik, S. N., Li, D., El-Baz, K., Shalaby, M., Al-Karaki, R., Nasr, M., Klein, C. D., Abouzid, K. A. M. & Seleem, M. N. (2020). *Eur. J. Med. Chem.* **186**, 111850–111858. 10.1016/j.ejmech.2019.111850 31735572

[bb7] Dolomanov, O. V., Bourhis, L. J., Gildea, R. J., Howard, J. A. K. & Puschmann, H. (2009). *J. Appl. Cryst.* **42**, 339–341.

[bb8] Dvornikova, I. A., Buravlev, E. V., Fedorova, I. V., Shevchenko, O. G., Chukicheva, I. Y. & Kutchin, A. V. (2019). *Russ. Chem. Bull.* **68**, 1000–1005. 10.1007/s11172-022-3676-y PMC9762639 36569656

[bb9] El-Medani, S. M., Youssef, T. A. & Ramadan, R. M. (2003). *J. Mol. Struct.* **644**, 77–87.

[bb10] Fathima, K. S., Kavitha, P. & Anitha, K. (2017). *J. Mol. Struct.* **1143**, 444–451.

[bb11] Groom, C. R., Bruno, I. J., Lightfoot, M. P. & Ward, S. C. (2016). *Acta Cryst.* B**72**, 171–179. 10.1107/S2052520616003954 PMC4822653 27048719

[bb12] Low, J. N., Cobo, J., Abonia, R., Insuasty, B. & Glidewell, C. (2003). *Acta Cryst.* C**59**, o669–o671. 10.1107/s0108270103023333 14671365

[bb13] Mahendiran, D., Vinitha, G., Shobana, S., Viswanathan, V., Velmurugan, D. & Rahiman, A. K. (2016). *RSC Adv.* **6**, 60336–60348.

[bb14] McKinnon, J. J., Jayatilaka, D. & Spackman, M. A. (2007). *Chem. Commun.* pp. 3814–3816. 10.1039/b704980c 18217656

[bb15] Muthiah, P. T., Francis, S., Bocelli, G. & Cantoni, A. (2003). *Acta Cryst.* E**59**, m1164–m1167.

[bb16] Ottanà, R., Carotti, S., Maccari, R., Landini, I., Chiricosta, G., Caciagli, B., Vigorita, G. M. & Mini, E. (2005). *Bioorg. Med. Chem. Lett.* **15**, 3930–3933. 10.1016/j.bmcl.2005.05.093 15993594

[bb17] Ramla, M. M., Omar, A. M., Tokuda, H. & El-Diwani, H. I. (2007). *Bioorg. Med. Chem.* **15**, 6489–6496. 10.1016/j.bmc.2007.04.010 17643992

[bb18] Rigaku OD (2020). *CrysAlis PRO*. Rigaku Oxford Diffraction, Yarnton, England.

[bb19] Saiadali Fathima, K., Sathiyendran, M. & Anitha, K. (2019). *J. Mol. Struct.* **1177**, 457–468.

[bb20] Sheldrick, G. M. (2015*a*). *Acta Cryst.* A**71**, 3–8.

[bb21] Sheldrick, G. M. (2015*b*). *Acta Cryst.* C**71**, 3–8.

[bb22] Spackman, M. A. & Jayatilaka, D. (2009). *CrystEngComm*, **11**, 19–32.

[bb23] Spackman, P. R., Turner, M. J., McKinnon, J. J., Wolff, S. K., Grimwood, D. J., Jayatilaka, D. & Spackman, M. A. (2021). *J. Appl. Cryst.* **54**, 1006–1011. 10.1107/S1600576721002910 PMC8202033 34188619

[bb24] Suku, S. & Ravindran, R. (2023). *J. Mol. Struct.* **1283**, 135217–135228.

[bb25] Xiong, J., Hu, M.-L., Shi, Q. & Xiao, H.-P. (2003). *Z. Kristallogr.* **218**, 565–566.

[bb26] You, W., Fan, Y., Qian, H.-F., Yao, C. & Huang, W. (2009). *Acta Cryst.* E**65**, o115. 10.1107/S1600536808041706 PMC2968037 21581577

