# Supplementary material for: Synthesis, crystal structure, and Hirshfeld surface analysis of 1,3-di­hydro-2H-benzimidazol-2-iminium 3-carb­oxy-4-hy­droxy­benzene­sulfonate
Source: Acta Crystallogr E Crystallogr Commun. 2024 Sep 6;80(Pt 10):999–1002. doi: 10.1107/S2056989024008557 (PMC11451492; doi:10.1107/S2056989024008557)
Supplement: Supplementary file 3 [file e-80-00999-sup4.docx]

Table S1. Comparing of title compound with this literature (VITHIS: Fathima *et al.*, 2019; GAZTEI : Atria *et al.,*2012; EMIHAJ: Low *et al.*, 2003)

| **Bond length and Angels** | **Title compound** | **VITHIS** | **GAZTEI** | **EMIHAJ** |
| --- | --- | --- | --- | --- |
| N1─C6 | 1.389 (2) | 1.401 (2) | 1.390 (5) (N1-C38) | 1.391 (3) (N3-C7) |
| N1─C7 | 1.3389 (19) | 1.343 (2) | 1.351 (8) (N1-C39) | 1.350 (2) (N3-C1) |
| N2─C5 | 1.394 (2) | 1.400 (2) | 1.385 (7) (N2-C33) | 1.401 (2) (N2-C2) |
| N2─C7 | 1.335 (2) | 1.351 (2) | 1.352 (5) (N2-C39) | 1.341 (3) (N2-C1) |
| N3─C7 | 1.322 (2) | 1.315 (2) | 1.299 (6) (N3-C39) | 1.317 (3) (N1-C1) |
| N2─C7-N1 | 109.10 (13) | 109.14 (15) | 108.4 (4) (N2-C39-N1) | 109.1 (2) (N2-C1-N3) |
| N3─C7─N1 | 125.51 (14) | 125.07 (15) | 126.5 (4 ) (N3-C39-N1) | 124.4 (2) (N1-C1-N3) |
| N3─C7─N2 | 125.39 (15) | 125.79 (15) | 125.1 (4) (N3-C39-N2) | 126.5 (2) (N1-C1-N2) |
