# Supplementary material for: Synthesis, crystal structure, and Hirshfeld surface analysis of 1,3-di­hydro-2H-benzimidazol-2-iminium 3-carb­oxy-4-hy­droxy­benzene­sulfonate
Source: Acta Crystallogr E Crystallogr Commun. 2024 Sep 6;80(Pt 10):999–1002. doi: 10.1107/S2056989024008557 (PMC11451492; doi:10.1107/S2056989024008557)
Supplement: Supplementary file 4 [file e-80-00999-sup5.docx]

Table S2. Analysis of short ring-interactions with *Cg*-*Cg* distances of title compound **1**.

| *Cg*(X) = Plane number X | Distance between ring Centroids, Å | Slippage, Å |
| --- | --- | --- |
| *Cg*1─*Cg*1 | 3.761 | 0.995 |
| *Cg*1─*Cg*3 | 3.509 | 0.474 |
| *Cg*2─*Cg*2 | 3.982 | 1.306 |
| *Cg*2─*Cg*6 | 3.709 | 0.896 |
| *Cg*2─Cg7 | 3.729 | 1.289 |
| *Cg*4─*Cg*6 | 3.608 | 0.730 |
| *Cg*4─*Cg*7 | 3.757 | 1.422 |

*Cg* is corresponding the following ring centroids: *Cg*1 (C8–C13) dark sphere; *Cg*2 (C22–C27) orange phere; *Cg*3 (N1/C5–C7/N2) green sphere; *Cg*4 (C1-C6) red sphere; *Cg*6 (N4/C19-C21/N5) black sphere *Cg*7 (C15-C20) magenta sphere.
