# Supplementary figures and images for: Synthesis, crystal structure, and Hirshfeld surface analysis of 1,3-di­hydro-2H-benzimidazol-2-iminium 3-carb­oxy-4-hy­droxy­benzene­sulfonate
Source: Acta Crystallogr E Crystallogr Commun. 2024 Sep 6;80(Pt 10):999–1002. doi: 10.1107/S2056989024008557 (PMC11451492; doi:10.1107/S2056989024008557)

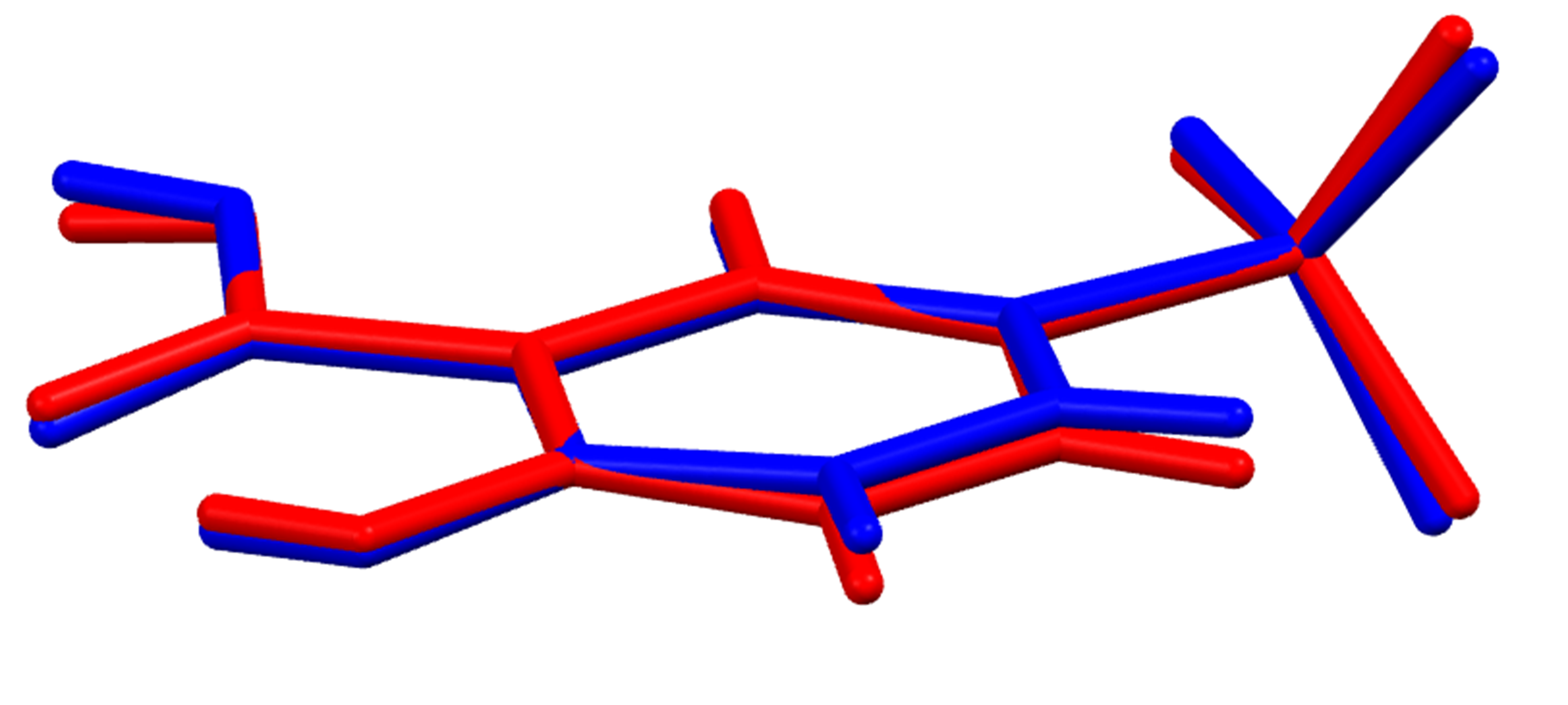

Supplement: Supplementary file 5 [file e-80-00999-sup6.tif]

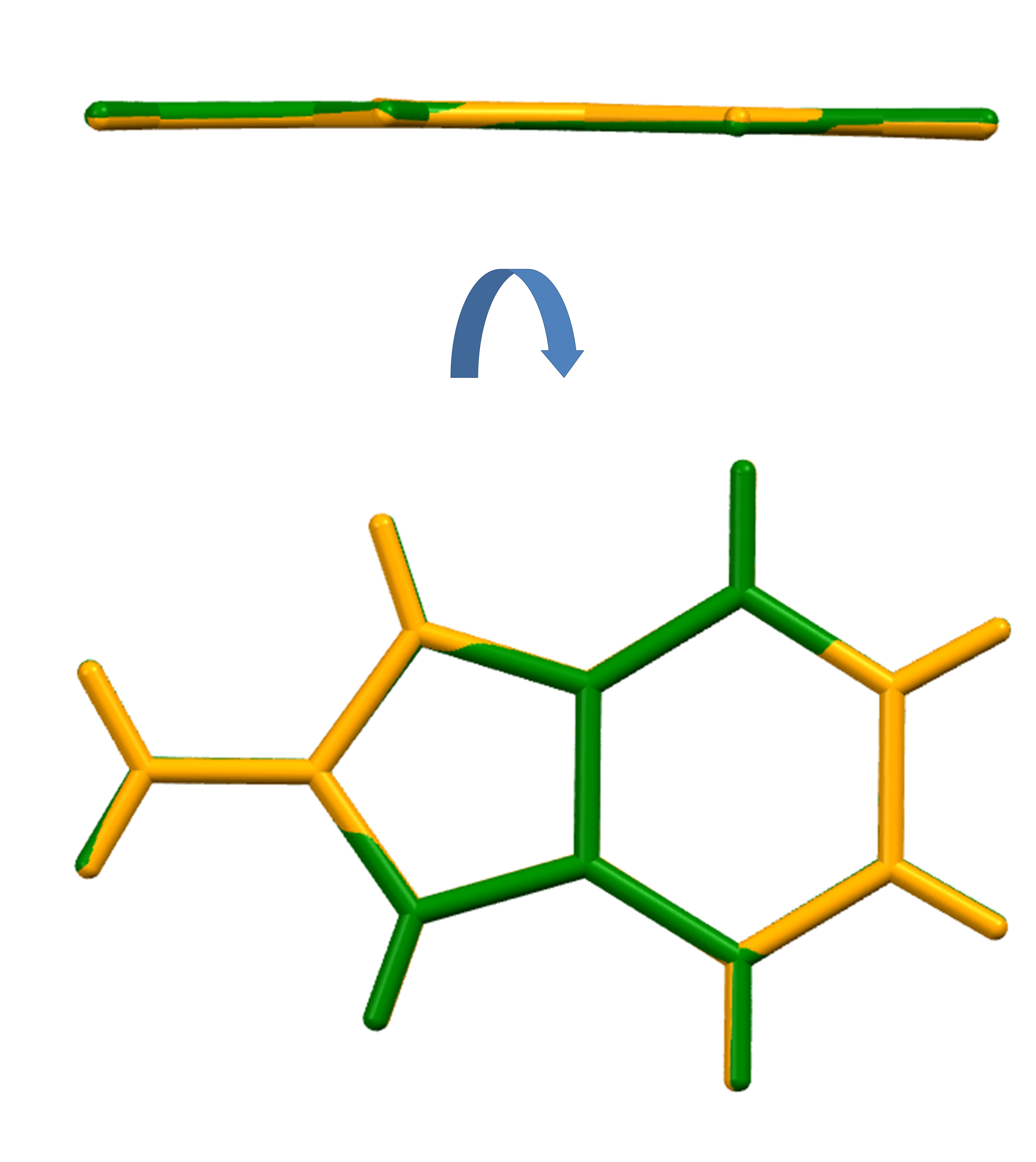

Supplement: Supplementary file 6 [file e-80-00999-sup7.tif]
